# The Priorities of End Users of Emergency Department Electronic Health Records: Modified Delphi Study

**DOI:** 10.2196/43103

**Published:** 2023-03-10

**Authors:** Matthew Yip, Alun Ackery, Trevor Jamieson, Shaun Mehta

**Affiliations:** 1 Temerty Faculty of Medicine University of Toronto Toronto, ON Canada; 2 The Institute for Health Policy, Management, and Evaluation University of Toronto Toronto, ON Canada; 3 Unity Health Toronto Toronto, ON Canada; 4 Department of Emergency Medicine North York General Hospital North York, ON Canada

**Keywords:** Delphi, EHR, electronic health record, emergency medicine, emergency, functionality, health information exchange, health system, medical informatics, patient-physician relationship, usability

## Abstract

**Background:**

The needs of the emergency department (ED) pose unique challenges to modern electronic health record (EHR) systems. A diverse case load of high-acuity, high-complexity presentations, and ambulatory patients, all requiring multiple transitions of care, creates a rich environment through which to critically examine EHRs.

**Objective:**

This investigation aims to capture and analyze the perspective of end users of EHR about the strengths, limitations, and future priorities for EHR in the setting of the ED.

**Methods:**

In the first phase of this investigation, a literature search was conducted to identify 5 key usage categories of ED EHRs. Using key usage categories in the first phase, a modified Delphi study was conducted with a group of 12 panelists with expertise in both emergency medicine and health informatics. Across 3 rounds of surveys, panelists generated and refined a list of strengths, limitations, and key priorities.

**Results:**

The findings from this investigation highlighted the preference of panelists for features maximizing functionality of basic clinical features relative to features of disruptive innovation.

**Conclusions:**

By capturing the perspectives of end users in the ED, this investigation highlights areas for the improvement or development of future EHRs in acute care settings.

## Introduction

Modern electronic health record (EHR) systems face difficulties meeting the unique needs of the emergency department (ED) [1-3]. High volumes of patients through the ED drive documentation burden; high-acuity cases demand efficient deployment of care measures; diagnostic uncertainty increases the need for clinical decision support tools; and the interdisciplinary, collaborative environment drives a need for EHRs to support efficient transitions of care [4]. In addition to these challenges, changes to the field of emergency medicine over the last several decades increase the need for highly efficient and capable information systems. As the complexity of patient’s presentations to the ED increases, measures of departmental crowding rise [5]. Complexity and nuance to treatment plans further increase need to leverage digital health tools in the management of complex patients to improve clinical decision-making and patient outcomes, albeit with increasing complexity of our digital systems [6-8]. The current COVID-19–mediated health human resource crisis has only exacerbated these challenges.

The International Standards Organization defines usability as “the extent to which a product can be used by specified users to achieve specified goals with effectiveness, efficiency, and satisfaction in a specified context of use” [9]. In the context of the ED, the specified goals of end users of an EHR may take on a variety of perspectives, given the different demands of this clinical space. A study evaluating the user-centered design principles of 11 EHR developers found that more than half of the developers had limited to inadequate interactions with clinicians in the development process of their products [10]. Despite the complexity of the unique needs of an ED EHR, there is a gap in the literature examining the perspective of the end user in an emergency medicine setting.

Delphi methods are a validated survey method to establish consensus opinion from a panel of experts [11]. The traditional Delphi process involves 3 rounds of information gathering: an initial round consisting of open-ended, qualitative questions followed by 2 rounds of Likert-scale rankings that allow for relative prioritization [11]. This process may be modified by introducing an initial set of parameters to narrow the scope of discussion [12-14]. A modified Delphi method offers the benefit of allowing focused discussion around specific attributes of a given problem. Delphi methods are unique in their ability to handle mixed types of information, both qualitative and quantitative in nature. They have previously been employed in the emergency medicine setting across several areas of investigation: investigating role definition of allied health team members, the development of violence screening criteria, the establishment of violence reduction strategies, and the selection of key performance indicators [15-18]. Delphi methods offer a validated method of synthesizing diverse perspectives about the current state and future improvements to ED EHRs.

To support hospital systems and practitioners develop future procurement criteria, and prioritize modifications, additions, or upgrades to their existing EHRs, we completed a systematic assessment of end user needs and priorities in the ED. This study aims to understand the nuances of perspective in physician end users regarding the ideal ED EHR.

## Methods

### Identification of Key Usage Categories

In phase 1 of our study, 2 independent reviewers completed review of academic literature on MEDLINE to build a list of usage categories of EHR. The reviewers also searched gray literature through web-based hand searches for topics related to information systems in acute care settings. After an iterative review of literature relating to both emergency medicine settings and EHR, 5 usage categories were developed inductively by the 2 reviewers. The findings were discussed with a working group comprised of 4 investigators with expertise in emergency medicine, health systems, and health informatics. The working group came to an agreement about 5 proposed key usage categories that were inputted into phase 2 to narrow the focus of discussion.

### Establishing Group Consensus Through Delphi Methods

Phase 2 used Delphi methods that involved sequential rounds of survey and data dissemination to experts in both emergency medicine and information systems regarding their perspectives on each of the 5 usage categories. Recruitment of expert panelists was done through purposive sampling beginning with 4 investigators identifying candidates with expertise in both the clinical environment of the ED and health informatics at 6 tertiary- and quaternary-care centers across southwestern Ontario, including 3 level 1 trauma centers. Subsequently, the identified candidates were also invited to provide information on other potential informants. In total, 12 expert panelists were recruited across several hospital systems with extensive experience in both emergency medicine and health information systems. The panelists were spread across 3 separate disciplines (7 of 12 in emergency medicine, 3 of 12 in pediatrics emergency medicine, and 2 of 12 in general internal medicine). Several panelists held multiple leadership roles in their departments, with 4 of 12 acting as either chief or deputy chief, 7 of 12 acting as department lead across roles in quality and safety, virtual care, artificial intelligence and machine learning, and quality improvement. Several panelists also performed adjacent clinical duties with 4 of 12 serving as Trauma Team Leaders. Two panelists also fulfilled C-level positions at their respective hospital systems for roles in medical informatics. All panelists were associated with the University of Toronto in teaching and academic roles.

The Delphi study was conducted in 3 rounds of surveys [11]. Survey administration was conducted using the Research Electronic Data Capture (REDCap 12.0.29) tools hosted at the University of Toronto [19,20]. To reduce bias in both survey responses and response analysis, the identity of all panelists was kept anonymous through the Delphi rounds. Panelists and investigators were unaware of the identity of panelist’s responses and panelists were not aware of the identity of other members of the Delphi panel until the conclusion of the study. The analysis of outputs from each round was conducted by 2 independent reviewers and consensus was established before circulation of findings to panelists between each round.

The first-round survey involved qualitative information gathering through free-text responses. Free-text responses were analyzed using NVivo (NVivo Version 12). First, responses were coded deductively, using usage categories defined in Phase I of the study. Second, sentiment coding was performed by NVivo’s sentiment analysis with manual adjustment and necessary recoding based on consensus by the 2 independent reviewers. Outputs were circulated to panelists for review. The second-round survey gathered quantitative information on the perceived importance of first-round outputs using Likert scales and qualitative free-text responses about areas of disagreement from first-round responses. The quantitative outputs from the second-round survey were analyzed using Microsoft Excel (MSO Version 2205; Microsoft Inc) to generate descriptive statistics around measured variables and the qualitative outputs from the second-round survey were circulated to the panelists [21]. The third-round survey focused on establishing a ranked list of priorities based on the second-round outputs with the highest perceived importance resulting in a ranked list of priorities for each usage category.

### Ethical Considerations

Phase 2 received the approval of the Research Ethics Board through the University of Toronto (protocol #00040996).

## Results

**Figure 1:**
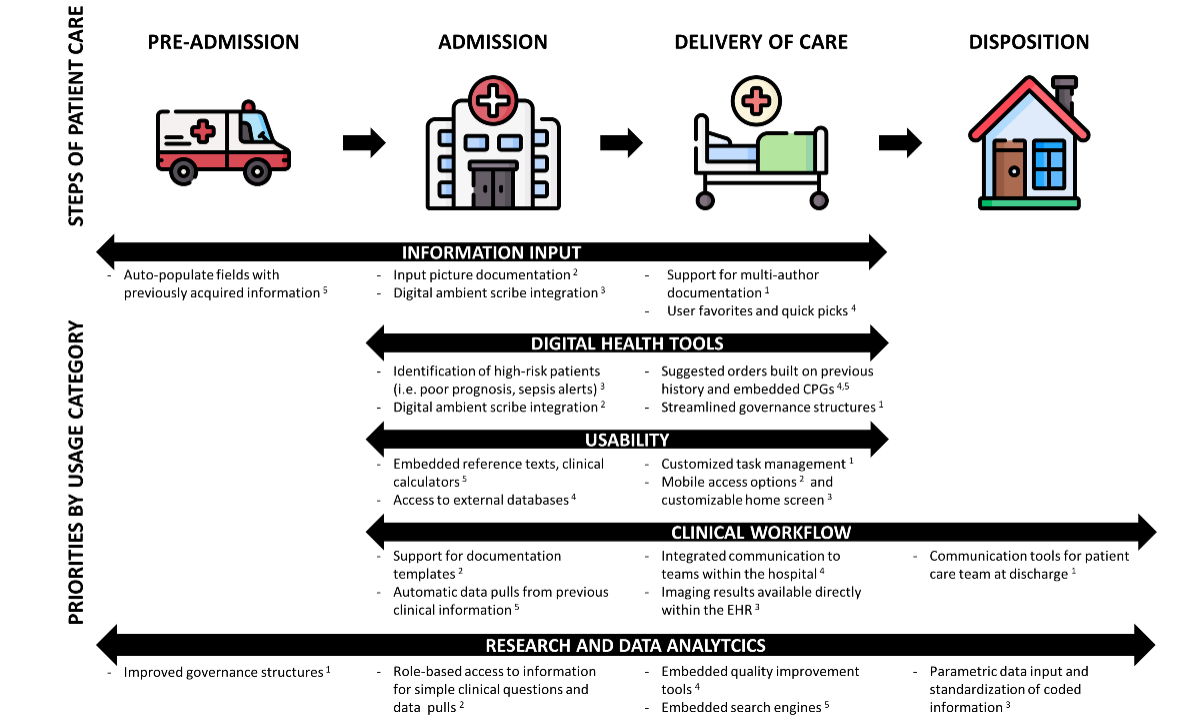
A conceptualization of the intersection between the ranked priorities of panelists by usage categories and steps of the patient care journey. Innovations below each category are informed by Delphi outputs and offer a nonexhaustive view of proposed innovations across usage categories, affecting each step of care. Superscript after each priority denotes relative prioritization by panelists in Delphi rounds (1=highest priority, 5=lowest priority).

In total, the perspectives captured by the expert panel spanned 6 separate hospital sites and 5 separate EHRs. Across all 3 rounds of survey, there was full retention of the original cohort of 12 expert panelists with no loss to follow-up between rounds. By using 5 key usage categories established by the working group members in phase 1 of the project (Table 1), the first round of surveys gathered free-form responses about the current needs of each category and generated a list of 10 features per usage category for a total of 50 features. Through the second-round survey, the panelists narrowed down the list to 25 features across key usage categories. Finally, in the third round of the survey, the panelists prioritized the top 5 features in each usage category relative to one another, for a total of 25 priorities (Textbox 1). Analysis of free-text responses produced statements of strengths and weaknesses for each category (Table 2). Several panelists raised ideas that may fall under the term of potential disruptive innovation, defined by Clayton Christensen as, “an innovation that makes things simpler and more affordable, and ‘technology’ is a way of combining inputs of materials, components, information, labor, and energy into outputs of greater value” [22]. Based on the priorities defined in Textbox 1 and the free-text responses by Table 2, possible features and innovations have been mapped to a typical journey through the ED, as a conceptualization of what an EHR may look like with these suggestions implemented (Figure 1).

**Table 1:** Usage categories defined by literature review.

Usage category	Definition	Example
Information input	The methods by which patient information is added or modified by care providers through multiple mediums [23-27]	Mobile device access, dictation support, and multidisciplinary access
Digital health tools	Features that augment or streamline the provision of care by providers [28-30]	Clinical decision support and computerized physician order entry
Usability	The extent to which a product can be used by specified users to achieve specified goals with effectiveness, efficiency, and satisfaction in a specified context of use [10,31-36]	Personalized dashboards, customizable quick picks within order sets, and inbox and task management
Clinical workflow	EHR^a^ features that impact patient flow through the ED [37-42]	Multidisciplinary communication and tools for communicating with external care providers after a visit to the emergency department
Research and data analytics	EHR features that allow for the ability to investigate research questions or conduct quality improvement studies [43-45]	Artificial intelligence and machine learning algorithms or adherence to interoperability standards

^a^EHR: electronic health record.

**Key priorities defined by Delphi outputs (1-5 to indicate their priority with 1 being the highest and 5 being the lowest).**

**Information input**

1. Support for multiauthor documentation
2. Include the ability to input picture documentation
3. Integrate digital ambient scribes to expedite note taking
4. Enable quick picks or user favorites for easily accessed orders
5. Auto-populate fields with information that has already been given during the visit (ie, triage assessment, consults from other services) or already available (ie, past visits or community health record databases)

**Digital health tools**

1. Streamlined governance structures to support pushing and pulling data from an electronic health record (EHR)
2. Integration of digital ambient scribes to expedite documentation time and order set suggestions
3. Identification of high-risk patients (ie, poor prognosis and sepsis alerts)
4. Order entry and clinical decision support that builds on existing history for a given patient and continues to build on this history for subsequent visits
5. Embed clinical tools such as clinical practice guidelines or common risk stratification tools

**Usability**

1. Improve inbox and task management within EHR by allowing users to customize layout of their inbox
2. Streamline mobile access options that prioritize information input, similar to eCommerce or food delivery applications
3. Implementation of customizable home screen
4. Streamline access to other sources of information (ie, community health record databases and previous medication reconciliations)
5. Streamline the number of required systems for different tasks or minimize disruption to workflow through improved integration

**Clinical workflow**

1. Support of patients beyond the hospital setting such as discharge instructions with prescriptions sent to an email or via SMS
2. Support for uploading documentation templates
3. Access imaging results within the EHR
4. Ability to communicate with others both inside the hospital setting (ie, paging consults, porter services, and housekeeping) and beyond the hospital setting (ie, community physicians, and emergency medical services)
5. Automatic data pulls from previous clinical documentation rather than manual chart review

**Research and data analytics**

1. Improved governance structures that afford more flexibility to the end user with respect to access
2. Increase information access using role-based access (ie, quality improvement lead, chief, and research roles), allowing for expedited data pulls and enabling queries for simple questions
3. Enhance standardization of coded information (ie, diagnosis, chief complaints, and patient outcome) within sites and across sites
4. Embedded quality improvement tools
5. Embedded search engines to query and trend simple questions

**Table 2:** Strengths and limitations by category.

	Strengths	Limitations
Information input	Improved accuracy of information in charting Improved collation of information and documentation for the overall care journey of a patient Support for verbal dictation methods expedites documentation	Charting demands of EHRs^a^ increase documentation burden Redundancy of information input is attributed to the inability to carry over information previously gathered in the visit Some EHRs do not support all information formats (ie, pictures, ECGs)
Digital health tools	Order sets have increased the ease of use and safety is increased by decision support teams Current digital tools support patient safety EHRs have the technical capacity for deployment of innovative digital health tools, despite logistical difficulty and limitation of available health human resources	Balancing innovative technology (ie, artificial intelligence, machine learning, and natural language processing) with patient safety, impossible to “try fast, fail fast” in the ED^b^ environment Governance structures such as privacy rules around information ownership, access rules within the department, limit accessibility of information
Usability	Changes to order sets undergo a strict process to ensure that changes are in keeping with best available evidence Note templates are helpful in reducing documentation burden	Standardization ensures patient safety but compromises flexibility of EHR “Look and feel” modifications are difficult to make with current systems Inbox and task management customization is not widely available
Clinical workflow	EHRs effectively collate information from past visits and current visit Makes interprofessional care between physicians, nurses, and clericals more seamless Data entered are more accessible and more legible	Redundancy of gathering information and reinputting slows workflow Multiple systems are required for clinical tasks (ie, imaging results and past visits) Documentation burden reduces face-to-face time
Research and data analytics	EHRs support data organization Increased ease of coding information in electronic form Supports a surplus of information relative to what is used	Access to information is limited by privacy rules Steps of procedure for access to information for research is cumbersome, even for basic information or search queries Quality of information stored in the EHR due to lack of parametric data storage (ie, dropdown menus for diagnosis, checkboxes for signs, and symptoms)

^a^EHR: electronic health record.

^b^ED: emergency department.

### Category I: Information Input

Overall, it was found that panelists preferred that current EHRs improve on existing capabilities before trying to tackle potential disruptive innovations [22]. Panelists specifically listed and ranked digital ambient scribes, which process information from a patient–physician interview into a note in an attempt to reduce documentation burden, and auto-population of documentation from other sources of clinical information, lower than basic functionality such as multiauthor documentation and support for documentation of other forms of media. As strength, it was found that panelists thought that EHRs have streamlined the collation and standardization of information. A limitation of current information input capabilities of EHRs is the lack of support for multiauthor documentation, increasing the need to repeatedly gather, and document redundant information that has already been collected by other members of the patient’s care team. This drives documentation burden and creates inefficiencies.

### Category II: Digital Health Tools

It was largely believed by panelists that human factors limit the implementation of digital health tools such as machine learning algorithms that provide clinical decision support, as opposed to the technical capacities of the current EHRs. Furthermore, the priorities list shows that panelists prioritize tools supporting clinicians in acute care settings such as identifying high-risk patients as opposed to pulling previous information from other sources such as previous charts or clinical portals. Panelists mostly expressed that EHRs have streamlined the ability to conduct repetitive, previously tedious tasks. However, they state that innovation requires large amounts of coordination and health human resources, so while the potential may exist for implementation, there may not be the current appetite or means to sustain this change.

### Category III: Usability

The priorities of end users in this category saw 2 sentiments of thought, which first may seem conflicting. On the one hand, there was an interest in having increased customizability options within ED EHRs, such as the enablement of customization of quick picks and inbox management. However, there was also the argument for adaptation on the part of the end user to the features and limitations of the EHR. Overall, panelists believed that EHRs have increased standardization of care delivery through order sets that are vetted by central decision support teams, ensuring that orders are up to current care standards. However, in their current form, EHRs are limited in the customization options that they provide for their end users, even with respect to personal workflow features such as inbox task management, or “look and feel” customizations such as the layout of a given dashboard.

### Category IV: Clinical Workflow

Panelists again prioritized basic functionality (ie, discharge planning, interdisciplinary communication) as opposed to disruptive innovation. Although EHRs have increased ease of collaboration among teams in the ED through collation of documentation from triage, panelists still raised concerns around the limitations of interoperability between hospital systems and other systems such as primary care EHRs. Additionally, even within a single-hospital system, it was found to be difficult to communicate with other services that did not use the same EHR or charting method (ie, different clinical systems or paper charting).

### Category V: Research and Data Analytics

Overall, panelists express that there was limitation with fluid access and usability of information. An undeniable strength of the EHR is that it has augmented the ability to collect, store, and access structured data. However, panelists identified that the ability to access the data in a meaningful way is still limited due to the format of stored data. Although it is possible to access volumes of information, the standardization of information input is lacking, such that any information sought for research purposes will still require manual recoding. Suggestions in this realm included improving drop-down menus to provide standardization of documentation input.

## Discussion

### Principal Results

The key usage categories developed in our investigation and the panelists’ priorities determined by Delphi outputs span several steps of a patient’s journey through the ED (Figure 1). These priorities highlight the balancing act that must occur in each usage category with the development and deployment of ED EHRs. With respect to information input, support for multiauthor documentation helps to reduce redundancy of information gathering and input, and support for innovations helps reduce documentation burden. With respect to digital health tools, improved governance structures could support the development and deployment of innovations that may aid in decision-making. With respect to usability, an optimized EHR for the ED would have customizability options for workflow and maintain strong standardization for deployment of care, such as order sets. With respect to clinical workflow support for communication beyond the hospital helps to ensure efficient and safe patient discharges, while consolidated information systems ensure efficient access to conducted investigations. With respect to research and data analytics, improved accessibility allows for more contribution from end users with respect to the development of new knowledge and useful clinical insights.

In Gawande’s [46] article titled, “Why Doctors Hate Their Computers,” Gawande writes of EHRs: “I’ve come to feel that a system that promised to increase my mastery over my work has, instead, increased my work’s mastery over me.” His assertion mainly centers around the collection of large amounts of unused information from a patient–physician encounter, which drives documentation burden and decreases patient–physician interaction time. Previous studies have estimated that the ED physicians may spend as much as 25% of the total time caring for a single patient on documentation [47]. Aligned with the previous literature and clinical experiences of documentation burden, Gawande highlights a key issue where EHRs can decrease efficiency and become a burden rather than a valuable tool.

These concerns are aligned with the findings from this investigation, with panelists broadly prioritizing functionality over disruptive innovation, and issues such as interoperability and the reduction of documentation burden being prioritized across several usage categories. For example, with respect to information input, support for multiauthor documentation and picture integration was prioritized over features such as digital ambient scribes or population from past documentation. Another example is seen in panelists’ priorities with respect to clinical workflow, where panelists prioritized discharge communication methods over auto-population of patient information from previous documentation. Panelists were sampled from a variety of care settings employing several different EHRs at each site, suggesting that no single EHR vendor comprehensively captures the priorities identified in this investigation.

By examining the discrepancies between the identified priorities of panelists and the qualitative responses of strengths and limitations, it is possible to identify areas for impactful improvements. For example, with respect to digital health tools, streamlined governance structures were identified as both a top priority (Textbox 1) and listed as a limitation (Table 2). Another usage category that demonstrated this was in research and data analytics, where panelists identified streamlined governance structures and increased role-based access as priorities (Textbox 1) and identified privacy as a limiting factor for gathering information (Table 2). Integrating this information identifies areas of high priority and can potentially inform prioritization of where system administrators can best optimize their own EHRs or build evidence-informed criteria in future acquisitions.

Compared to the deployment of Delphi methods in other emergency medicine clinical questions, the modifications to the process of this investigation optimized for depth of discussion in defined usage categories. The specific modification to the traditional process entailed defining the 5 usage categories through literature review which subsequently served as inputs to the Delphi model. Other investigations either integrate the literature review as one of the 3 traditional rounds or rely on free-text responses as a means to providing a focus of discussion [16,17]. A trade-off of the selected modification is that it prevents panelists from suggesting their own mental schema of usage categories of EHRs; however, this trade-off was made to achieve a deeper understanding of priorities within discrete categories. An additional benefit of a preliminary literature review is that focused discussion ensured concrete outputs from each round, which may have contributed to the complete retention of panelists across the 3 rounds of the Delphi process. Overall, through a preliminary literature review and a Delphi process with narrow targets based on prior inputs, the modified Delphi method strikes an appropriate balance between breadth and depth in the examination of ED EHRs.

### Limitations

One potential limitation to this study is the generalizability of findings. Panelists are familiar with both ED care settings and health informatics in tertiary-care hospitals in southern Ontario, all with enterprise-wide deployments of their hospital EHR. This may lead to panelist-specific prioritization of other clinically adjacent activities such as academic research or data organization. Subspecialty interests may introduce additional variance to captured perspectives. Furthermore, this investigation focused on capturing the perspectives of physicians as the end user, which does not capture the perspectives of other disciplines that engage with an ED EHR.

### Conclusions

Improving EHRs to effectively meet the unique priorities of the ED demands a thorough understanding of the priorities of end users. A modified Delphi approach allows an in-depth analysis of perspectives of expert panelists in discretely defined usage categories. Capturing the perspectives of an expert panel from tertiary and quaternary care centers across Southwestern Ontario and served by diverse EHR vendors, the findings of this study highlight end-user prioritization of functionality over disruptive innovation. At a provider level, these findings will lead to meaningful reflection and discussions with department leadership about how an EHR can fit local needs. At an institution level, these findings will have implications for choosing future EHRs and adaptation of existing systems. At a developer level, these findings will have further sensitized developers to the preferences of end users in high-acuity settings. The future steps in discussions around EHR improvement should involve gathering the perspectives of allied health professionals who also engage with EHRs and with patients as they are the beneficiaries of improvements to information systems. Furthermore, comparison of perspectives gathered in the ED to perspectives from other areas of the hospital would establish commonalities, common pain points, and enhance our understanding of the information system preferences of end users.

## Abbreviations

ED: emergency department
EHR: electronic health record
REDCap: Research Electronic Data Capture

## Data Availability

The data sets generated during and/or analyzed during the current study are available from the corresponding author on reasonable request.
